# Research on Prediction Model for Durability of Straw Bale Walls in Warm (Humid) Continental Climate—A Case Study in Northeast China

**DOI:** 10.3390/ma13133007

**Published:** 2020-07-06

**Authors:** Xunzhi Yin, Qi Dong, Mike Lawrence, Daniel Maskell, Jiaqi Yu, Cheng Sun

**Affiliations:** 1 School of Architecture, Harbin Institute of Technology, Harbin 150001, China; x.yin@hit.edu.cn (X.Y.); dongqi@hit.edu.cn (Q.D.); 19S034015@stu.hit.edu.cn (J.Y.); 2 Key Laboratory of Cold Region Urban and Rural Human Settlement Environment Science and Technology, Ministry of Industry and Information Technology, Harbin 150001, China; 3 Department of Architecture and Civil Engineering, University of Bath, Bath BA2 7AY, UK; m.lawrence@bath.ac.uk (M.L.); d.maskell@bath.ac.uk (D.M.)

**Keywords:** straw bale building, northeast china, durability, degradation assessment, experimental building, warm (humid) continental climate

## Abstract

This research analyses straw degradation inside straw bale walls in the region and develops the prediction of degradation inside straw bale walls. The results show that the straw inside straw bale walls have no serious concerns of degradation in the high hygrothermal environment in the region with only moderate concerns of degradation in the area 2–3 cm deep behind the lime render. The onsite investigations indicate that the degradation isopleth model can only predict straw conditions behind the rendering layer, whereas the isothermal model fits the complete situation inside straw bale walls. This research develops the models for predicting straw degradation levels inside a straw bale building in a warm (humid) continental climate. The impact of this research will help the growth of low carbon energy efficient straw bale construction with confidence pertaining to its long-term durability characteristics both in the region and regions sharing similar climatic features globally.

## 1. Introduction

Straw bale construction uses agricultural co-products in the building industry [1]. This building type was originally developed in Nebraska in the late 19th century due to a shortage of building materials [1]. The straw bale buildings were replaced after achieving more industrialised building materials in American mid-west in the early 20th century [2]. Due to the oil crisis in the 1970s, people developed ideas for creating more energy efficient buildings in the Western world. Straw bale buildings were characterised by their low cost, quick construction process, and high thermal insulation, and were treated as an effective build type to achieve a high standard of energy efficiency in the era [3]. The construction technique was re-introduced in the 1980s in west America and the construction method became popular worldwide later [4]. Straw bale construction has now become more recognised globally and has developed into a contemporary building typology. However, its development and implementation have largely been limited to European and American climates that, while varied, are considered mild. For a wider impact of this construction approach, an understanding of its performance in extreme environmental conditions, such as the severe cold region in China, is required.

**Figure 1:**
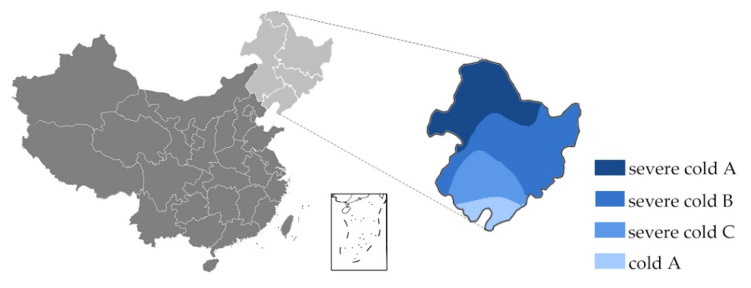
Climatic regionalisation of Northeast China (redrawn from [5]).

The climatic regions of China are classified by the Ministry of Housing and Urban-Rural Development (MOHURD) in the GB50178-93 [5]. In the standard GB50178-93, the regions of northeast China are classified as severe cold and cold regions (Figure 1). In the newly developed Köppen climate classification regarding the climatic features up to date, major regions of northeast China are identified as having a warm (humid) continental climate [6]. The climate region features monsoon-influenced hot and warm summer with humid continental climate [7]. In the region, the coldest month averages below 0 °C and the hottest monthly temperature averages over 22 °C with a dry winter and humid summer [7].

The construction technique was initially introduced to northern China by the Adventist Development and Relief Agency (ADRA) in 1998 [8]. The project was funded by the ADRA/China Central Government and local governments in northern China and more than 600 straw bale buildings were finished by 2006 [9]. However, recent research has shown degradation issues inside the straw bale walls in the project [10] and the current status of the straw bale buildings remains uncertain [11,12].

To assess the durability issues of straw bale construction in the warm (humid) continental climate, this research aims to analyse the straw degradation through both experiments using specimens of straw bale walls and the inspection of an experimental building constructed in northeast China. Therefore, the objectives are to develop proper predicting models for straw degradation regarding the warm (humid) continental climate and to expand the construction of straw bale buildings in wider regions globally. The research begins with both laboratorial research and onsite research of straw degradation following the analysis of predicting models for the degradation process in comparison with the laboratorial and onsite research results.

## 2. Background

### 2.1. Favourable Environment for Supporting Straw Degradation

There are two kinds of degradation processes within straw bale walls, namely aerobic degradation and anaerobic degradation [11]. The availability of oxygen for microorganisms inside straw determines what kind of degradation occurs. The presence of oxygen is an accelerator of straw degradation, with suitable hygrothermal environments leading to the readily aerobic decomposition of straw [13]. However, straw bale walls are commonly isolated from the external environment by a render layer and oxygen concentration can only support the initial aerobic degradation of straw [14].

Even though different types of rendering materials have various permeabilities for water and air transmittances, all the rendering approaches typically provide a relatively sealed environment for the straw bales [15]. The relationships between different render properties and the decomposition resistance of straw bales are not fully understood in existing research [16], as all rendering materials have the ability to limit moisture content and achieve similar airtightness inside straw bale walls [3]. This can gradually reduce oxygen level inside straw bale walls and therefore lead to the anaerobic degradation of straw inside walls [17]. In the process of anaerobic degradation, straw has a much longer decomposition duration than the aerobic decomposition. As more bacteria are needed oxygen to trigger the biological reaction which can change mineralogical nitrogen to proteins, the active microorganisms for anaerobic degradation are of low quantity in sealed straw bale walls [18].

The environment inside straw bale walls can protect straw from the hostile activities of microorganisms. Bacteria and yeasts can only duplicate in high moisture conditions, and other than this, yeasts also need light to process the biochemical reactions which are essential for the growth of microorganisms [19]. Considering the environment inside a rendered straw bale wall, a barrier between straw bales and the external air is formed. As a result, relatively dry with no light conditions are achieved, which cannot accommodate the growth of microorganisms [20]. However, there would be issues for straw bale walls with the presence of liquid water between the rendering and the straw bales [17]. The straw bale walls which are exposed to high humidity and a differential temperature on both sides may lead to condensation in the straw bales and therefore straw will be degraded by long time exposure to liquid water.

### 2.2. Predicting Models for Straw Degradation

The hygrothermal environment within the straw bale walls and absolute moisture content of straw are two critical factors that lead to straw degradation. As such, this section reviews the existing methods for predicting the critical hygrothermal within straw bale walls and the critical moisture content of straw for straw degradation.

**Figure 2:**
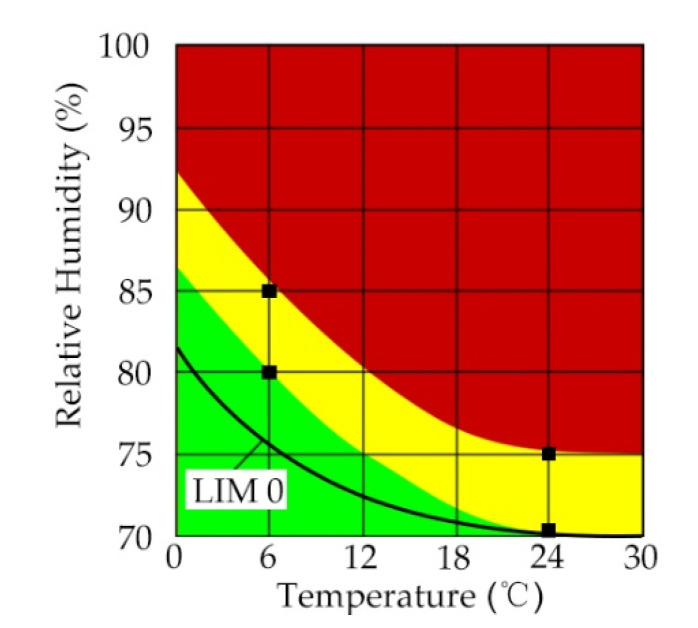
Isopleth system of wheat straw [22].

The isopleth system was designed to describe specific reactions from growth of different mould species in different hygrothermal environments [21]. By considering the degradation characteristics of building materials, an isopleth was developed to help predict the severity of potential degradation of building materials due to differing hygrothermal conditions [22]. Sedlbauer et al. used 12 different incubation units with different hygrothermal conditions to evaluate the mould growth of different building materials for 100 days and categorised risks of mould growth in three colours (Figure 2): red colour presented high risks of mould growth, yellow colour indicated moderate concern of mould growth, and the green colour represented no decomposition risks of the examined building materials [22]. As the degradation isopleths were developed to predict mould growth on the surface of building materials with the oxygen levels of ambient air [22], the isopleths may be different for the degradation of straw within straw bale walls. In the research of straw degradation in the UK, due to the low-oxygen environment in the straw bale walls, straw was in a good condition in the environment which would have been classified in the red area in the degradation isopleth [14].

Key to understanding the potential degradation is an understanding of the relationship between the relative humidity, temperature, and the moisture content of the material. The sorption isotherm describes the direct relationship between the moisture content of hygroscopic materials and the surrounding RH environment [23]. Hedlin used a jacketed air bath to demonstrate the sorption isotherms for five different grain straw species [24]. The research examined different species of straw (oat straw, barley, and two types of wheat straw) in different relative humidity conditions at 21.1 °C [24]. The empirical equation of the moisture content of straw and surrounding humidity is as follow [24]:

(1) φ=1−Kc(1−CCs)1+[CsC−1n]ay3i

where:

φ = relative humidity

C = moisture content at relative humidity, φ

$C_{s}$ = fibre saturation moisture content (400%)

n = $C_{s}/C_{50\%RH}$

$K_{c}=$ 0.0227, and

i = 1.6

The equation cannot convert the surrounding RH environments to moisture contents without knowing air temperatures. The air temperature can change rapidly in straw bale walls in real conditions which results in a restricted use of the equation in predicting the moisture content of straw bales in walls. Strømdahl used controlled climatic chambers to investigate the water absorption properties of four plant fibres in different temperatures [25]. The sorption isotherm of wheat straw showed no significant differences from 20 °C to 60 °C [25]. Compared to the isotherms at 2 °C and 4 °C, the moisture content of wheat straw was slightly lower at 6 °C. Other than the research of Strømdahl, a separate research showed no significant differences of moisture content within the straw at temperatures between 5 °C and 35 °C [26]. The following research of Lawrence et al. [27] indicated that temperature differences had ignorable effects on sorption isotherm of straw and simplified the Hedlin [24] equation by ignoring the effects of temperature. The simplified equation had similar results in predicting moisture content of wheat straw [27]:

(2) C=Cs1+n(Kmφ−1)i3

where:

C = moisture content at relative humidity, φ

$C_{s}$ = fibre saturation moisture content (400%)

φ = relative humidity,

n = 44

$K_{m}$ = 1−$K_{c}$ = 0.9773, and

i = 1.6

Yin et al. [28] further developed this steady state relationship which was later modified through considering the dynamic effect of time to reach full moisture sorption of straw. The straw was placed in various RH environments for 24 h which was designed to reach the unsaturated conditions of straw [28]. Due to the relatively short term of exposition of straw in various RH environments in the experimental conditions, straw does not reach moisture stability until the end of 24 h. Based on the experimental results, the unsaturated isothermal model changed the constant ‘*n*’ from 44 to 54 to indicate a closer prediction of the sorption isotherm of straw in diurnal situations [28].

## 3. Method

To assess the possibility of degradation induced by the high RH and high temperature conditions, an experimental investigation was designed to represent a typical wall build-up. The experimental results will be compared with onsite visits of one experimental building constructed in Changchun to access the degradation potential of straw within straw bale walls in northeast China.

### 3.1. Climatic Condition of Examined Area

**Figure 3:**
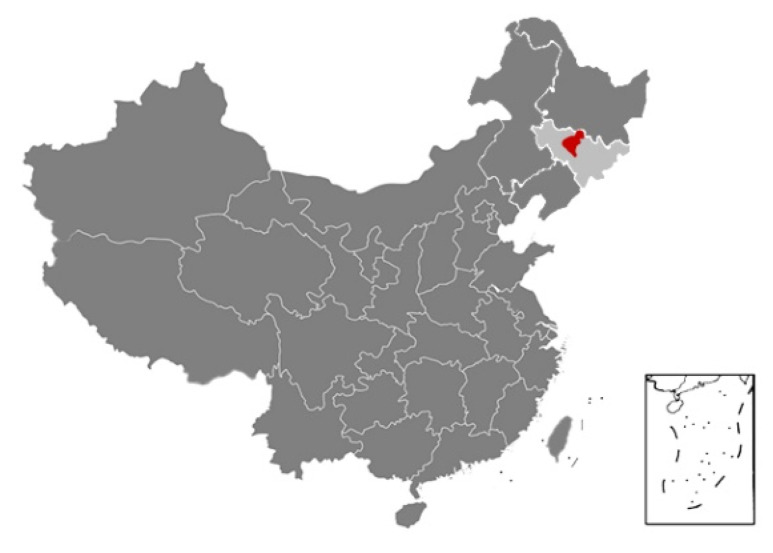
Location of Changchun in China.

Changchun is the capital city of Jilin province (Figure 3) and the whole province is identified in the severe cold climate region in the standard [5]. Temperature peaks at around 30 °C in the area from June to August and drops to below freezing after late October annually. The highest monthly air humidity level is 88% and it appears in January.

The monthly humidity levels are from 63% to 72% in summer during which the highest temperature appears. The climatic features of Changchun represent three typical climatic characteristics in the severe cold region in China: Firstly, both the air temperature and humidity are at high levels in summer months. The daily high temperatures are around 30 °C in the summer months. Due to the features of the warm (humid) continental climate, rainfalls are expected mostly in summer months which lead to high air humidity. Secondly, as the lowest rain potentials and air humidity are expected in spring months, the spring can be identified as a “dry” season in northern China. However, as air temperatures begin to rise above freezing in March, melting snow increases humidity levels in the severe cold region. Comparing the air humidity levels in April and May, the monthly average humidity of March is significantly higher than the one in April and May. Thirdly, the air temperatures are expected to be below freezing during the whole winter months in the severe cold region and the highest monthly air humidity levels present during the same period. In contrast, the high air humidity levels do not result in the humid environment inside and outside buildings [29]. As the low temperatures decrease absolute water vapour pressures in the air, the relative humidity levels in winter months are significantly higher than other months annually in the severe cold region. As a result, the winter months remain cold and dry in the severe cold region of China [5].

### 3.2. Conditions within Experimental Building

An experimental straw bale single-story bungalow with a double pitched roof was constructed in the southeast of Changchun (43°47′29.5″ N, 125°24′26.4″ E). The layout of the building is similar to the existing residential building in rural regions of Northeast China. The structural frame and foundation of the experimental building are made of steel reinforced concrete, which is conventional in the area familiar to local builders. For the same reason, the building is designed to have a cold roof, thus the insulation layer in the roof is laid beneath the roof frames and above the ceiling. The experimental building is constructed in an open field on the campus of Jilin Jianzhu University. The building is oriented on cardinal directions and there are no structures or other obstructions around the building. As the building is for experimental purposes only, there is no heating system included in the building.

**Figure 4:**
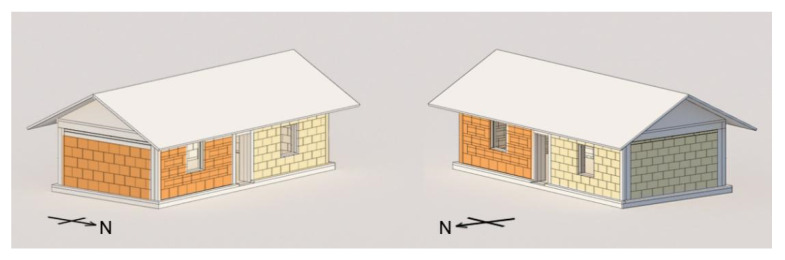
Perspective of the experimental building with laid flat straw bales (**light yellow**) and on edge straw bales (**dark yellow**).

There are two bale stacking methods used to examine the impact of straw orientation has on various properties, including durability and hygrothermal variation within the experimental building. The west flank of the building has laid-flat straw bales and the on-edge bales are used in the east flank (Figure 4). Due to dimensional variation, the laid on-edge walls have one less course of bales compared to the laid flat straw bale walls. To minimise thermal bridging of the concrete structure, Expanded Polystyrene (EPS) insulation boards were used between the concrete frames and between the straw bales and the frame in the east gable wall and the west gable wall. The experimental building was constructed from April 2016 and finished in July 2016. The processes of visual inspection of the straw conditions were conducted one year after the completion of the experimental building (19th June 2017). During the onsite research, lime render layer was opened to check the straw status within straw bale walls. Moisture contents of the straw within walls were measured directly using Balemaster (GRN6165) moisture content meter (Protimeter, Taunton, UK).

### 3.3. Small Scale Controlled Conditions

The investigation of the degradation potential of straw uses a climate-controlled chamber to replicate the summer hygrothermal environments in Changchun. The model of the climatic chamber is DY110(C) (Angelantoni Climatic Systems S·P·A (ACS, Marignano, Italy) and can control the temperature to ±0.1 °C. Makhlouf et al., demonstrated the importance of measuring materials under representative environmental conditions within the lab [30], so the conditions of the climatic chamber were set up at 95% RH and 35 °C to represent the potential peak daily mean temperature and peak daily mean RH. A 12-week duration of testing was used as it is slightly longer than the summer months in Changchun. The duration includes an allowance for an initial 2 weeks for moisture build up within the sealed straw bales and the following 10 weeks replicate summer months with higher hygrothermal environments than the situations in the severe cold region.

**Figure 5:**
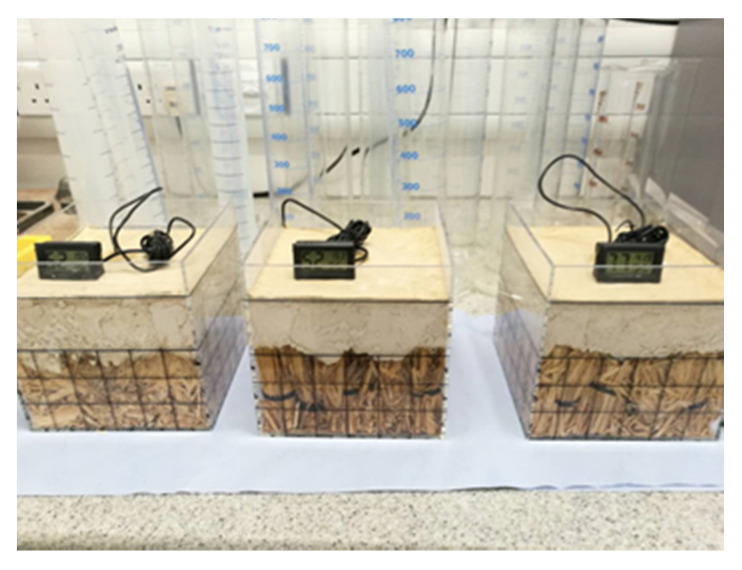
Section of walling construction in the experiment of small-scale controlled conditions with parallel aligned rice straw (**left**), perpendicular aligned wheat straw (**middle**) and perpendicular aligned rice straw (**right**).

**Figure 6:**
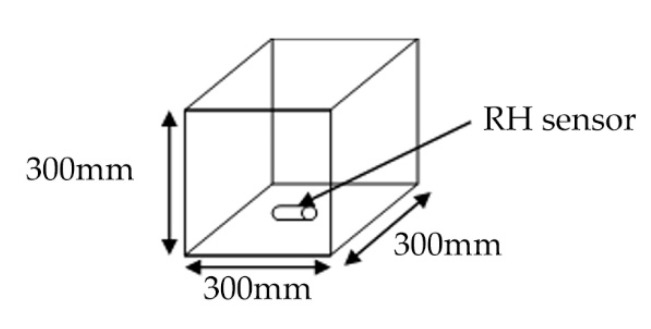
Diagram of experiment specimen and sensor set up.

Straw bales and lime render are constructed in three transparent boxes to replicate walling constructions of straw bale buildings (Figure 5). The rice straw used in straw bale buildings was examined and the wheat straw was examined for comparison with the existing knowledge of straw degradation as well. The straw is baled in small bundles and placed both in parallel and perpendicular to the lime render to replicate the laid flat stacking method and the laid on-edge stacking method in typical constructions of straw bale buildings. RH/T sensors (Dalian RHsens Technology Co., Ltd., Dalian, China) are installed inside the straw bundles in all the specimen (Figure 6). The thickness of lime render is 45–50 mm with the adjacent area between the lime render and the transparent boxes sealed with wax to avoid direct pass of moisture traveling into the straw. The surfaces of the transparent boxes are divided into 50 × 50 mm small rectangles to identify possible areas of straw degradation.

The specimens were initially placed in a controlled climatic room (80% RH @ 20 °C) for one week to cure the lime render. The cured specimens are then placed in low temperature (40 °C) oven for one week to reach lower than 85% initial RH @ 30 °C before placing specimens in the climatic chamber. During the 12-week experimental period, the conditions of straw are visually checked once a week at the beginning of the four weeks and once a day for the following eight weeks.

## 4. Results

### 4.1. Small Scale Controlled Conditions

Due to different moisture adsorbing speeds of the straw, as demonstrated by Yin et al. [28], the lime render introduces different amounts of moisture in each specimen and the initial RH readings are different in the specimen. The initial RH readings of the specimens are 52% RH and 77% RH for the specimen with parallel aligned rice straw and perpendicular aligned rice straw, respectively. This behaviour is not observed for the wheat straw where 55% RH was measured for the specimen with perpendicular alignment. The different humidity levels can be attributed to the different adsorption processes through the external surface and cutting end of straw. Despite the notable difference of initial RH in the straw bundles, all specimens reach 95% RH within the climatic chamber. The over 95% RH levels are reached after nine days and 12 days for rice straw and wheat straw aligned perpendicularly, respectively. The specimen of parallel placed rice straw took 15 days to reach 95% RH, indicating the impact of the aligned nature of the straw.

**Figure 7:**
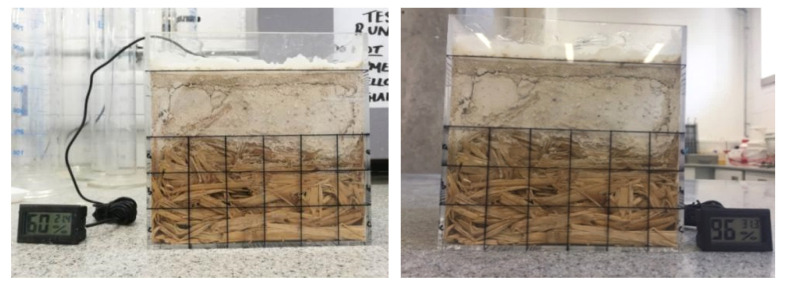
Specimen of rice straw with parallel placing before the degradation research (**left**) and after 12 weeks in climatic chamber (**right**).

**Figure 8:**
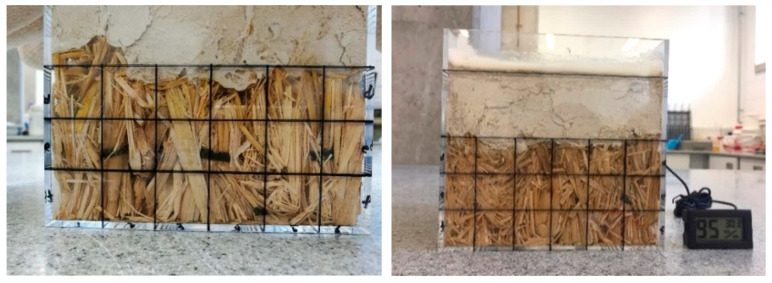
Specimen of rice straw with perpendicular placing before the degradation research (**left**) and after 12 weeks in climatic chamber (**right**).

**Figure 9:**
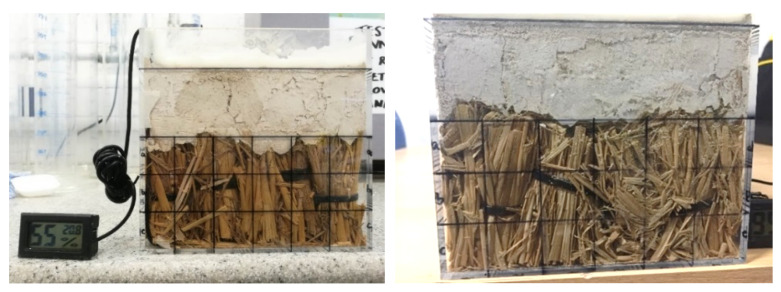
Specimen of wheat straw with perpendicular placing before the degradation research (**left**) and after 12 weeks in climatic chamber (**right**).

The visual checks of the specimens do not identify any recognisable straw degradation both during the experimental process and at the end of the experiments (Figure 7, Figure 8 and Figure 9). The surface colour of the straw is almost the same as it was before 12 weeks of experiments inside the climatic chamber. Existing research on the sorption isotherm of straw has identified that straw would have serious degradation after more than four weeks of exposure in the environment of 95% RH and 22 °C [27,31]. In contrast, the presence of the lime render layer creates the physical barrier and the straw is not exposed to high RH environment directly. Therefore, the lime render forms a protection layer for the straw and prevents serious degradation inside straw bundles. The reason for the lack of degradation may be because of oxygen availability and the presence of the high alkalinity lime render. The lime rendering provides a sealed environment which can limit the fungi and bacterial activity in the form of anaerobic degradation [11]. The anaerobic activity of fungi and bacteria can have slow degradation effects on the straw bales. Available free moisture is a crucial factor on anaerobic activity of microorganisms inside straw bale walls [32]. A lime wash is widely used in preventing mould growth on the walling surface in conventional buildings in China [33], and may result in similar effects when used for as a render on straw bale walls.

The experimental results show that straw does not exhibit signs of notable degradation in the potential high hygrothermal environment in the severe cold regions of China. The property of low degradation potential of straw at high temperature has specific importance in building straw bale constructions in the severe cold region. Due to the low degradation potential of straw in the high hygrothermal environments behind lime render layers in the region, the straw bales in the buildings can be constructed in the summer months with a similar degree of degradation in other months in the climatic region.

### 4.2. Results of the Experimental Building

**Figure 10:**
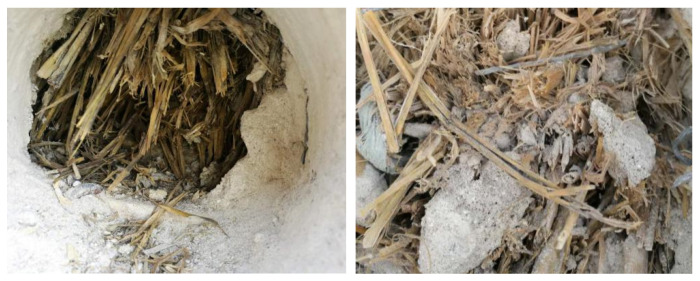
Degradation behind external render (**left**) and mixture of straw and lime render (**right**).

To examine the straw conditions behind the render in the experimental building, eight positions (Table 1) were examined to expose the inside of the straw bales. The external lime render of the eight positions was firstly removed following an examination of straw conditions behind the lime render. The following visual check of straw conditions identified a limited degradation of straw at the interface between straw bales and external rendering (Figure 10). Despite the differences between the eight examined positions, limited degradation was identified inside all the positions. The straw behind the interface remained a golden colour inside the walls. Unlike the limited straw degradation on the interface area between straw bales and the lime render, straw was in good condition with a mixture of the lime render (Figure 10). The site visit confirms the results of the laboratory research. The straw remained in a good condition both in the experimental conditions and inside the straw bale walls for one year.

**Figure 11:**
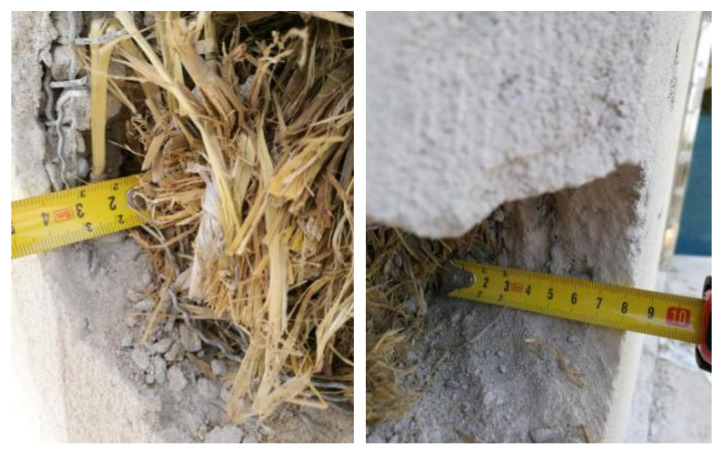
Straw conditions behind opening locations with thin rendering thickness (**left**) and thick rendering thickness (**right**).

From the onsite investigation, the thickness of the render did not visibly impact the conditions of the straw, with even the thinnest render (40 mm) seeming to be sufficient. The thickness of lime rendering varies significantly from the design thickness (Figure 11), with the thickness variations of lime rendering being between 40 mm to 105 mm in the experimental building (Table 1). The thicker lime render may provide a greater moisture buffering capacity to straw bales, but may have induced more initial moisture in the straw and resulted in a longer drying stage of both lime and the straw. However, the thicker lime render layers do not have a significant effect on straw degradation from the visual inspections.

**Figure 12:**
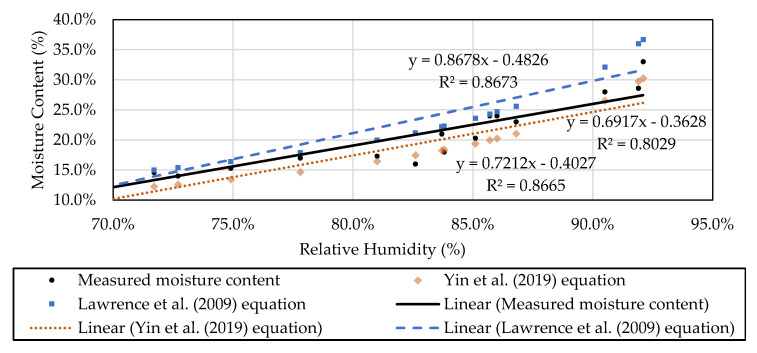
Comparison of calculated moisture content from isothermal model and actual moisture content in the experimental building.

The monitored results compare favorably to the isothermal models as presented by Lawrence et al. [27] and Yin et al. [28], with a sufficiently accurate estimation of moisture contents of straw bales inside walls predicted by these models. The calculated moisture content of straw through using existing isothermal models are compared to the measured moisture contents as measured in Table 2. The Lawrence equation shows an equal correlation between predicted and measured results (r value of 0.966) and, compared to Yin et al., (r value of 0.965), the Lawrence equation [24] would be less accurate with increasing monitored moisture contents and environment RH levels. As the monitored RH levels change rapidly in local areas, the saturated moisture contents of straw are seldom achieved regarding the climatic features in northeast China. Comparisons of the two existing isothermal models show that the unsaturated isothermal model can produce a closer prediction of the real moisture content of straw within walls in the climates of northeast China (Figure 12).

## 5. Assessment of Straw Degradation of the Experimental Building

**Figure 13:**
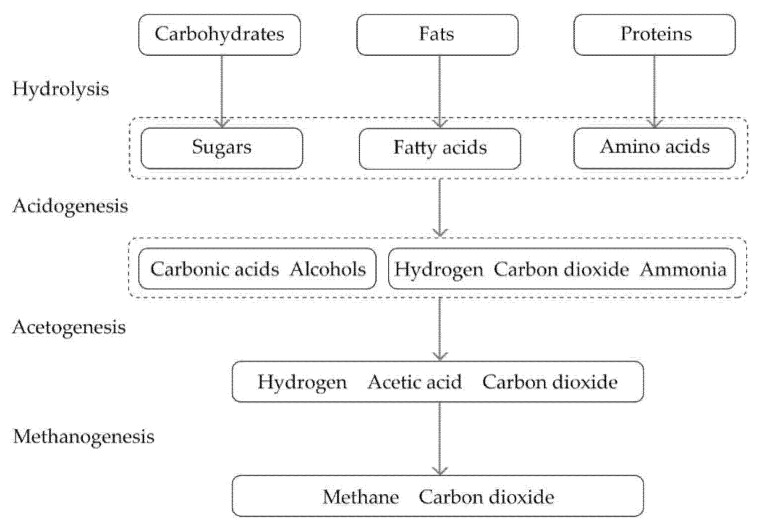
Stage pf anaerobic digestion.

While the overestimations of straw degradation by the isopleth model have been discussed in existing research, there has been little explanation for the inaccurate predictions of the isopleth model [14]. In this research, the presence of the lime render increases the durability of straw both in the laboratory under controlled conditions and in the filed under variable but realistic conditions. While there is limited understanding of the connection of anaerobic decomposition of straw and the rendering material, applications of lime render have observable effects on limiting anaerobic decomposition of straw in straw bale walls. During the anaerobic digestion process, carbohydrates in straw are initially converted into sugars in the hydrolysis stage [34]. The sugar is later broken down to intermediates (acetic acid, hydrogen, and carbon dioxide) by acidogenic and acetogenic bacteria. The methanogenic bacteria convert the intermediates into methane and carbon dioxide at the final stage of the anaerobic decomposition of straw (Figure 13).

The active PH range of the acidogenic and acetogenic bacteria is 6–10 and the methanogens have a smaller range of allowable pH range (7.5–8) [35]. With the presence of lime render, such a reaction would be limited for two reasons.

Firstly, the lime render would provide an unfavourable environment for the anaerobic decomposition of straw. The major content of lime is the calcium hydroxide which is a high alkaline pH material [36]. The pH of calcium hydroxide is over 12 which is notably over the active range of the acidogenic and acetogenic bacteria. During the curing stage of lime render, calcium hydroxide provides a long-term alkaline environment for straw within straw bale walls and therefore limits the decomposition of straw.

Secondly, the calcium hydroxide reacts with the intermediates of the anaerobic digestion of straw. Calcium hydroxide requires carbon dioxide in the chemical reaction of achieving calcium carbonate during the curing stage of lime based rendering and acetic acid is also neutralised by the high pH environment provided by the calcium hydroxide. As a result, the lime render reduces the intermediates of the anaerobic decomposition of straw and increasing the durability of straw bale walls. The effectiveness of lime rendering in increasing the durability of straw bale walls against anaerobic degradation remains uncertain [16].

**Figure 14:**
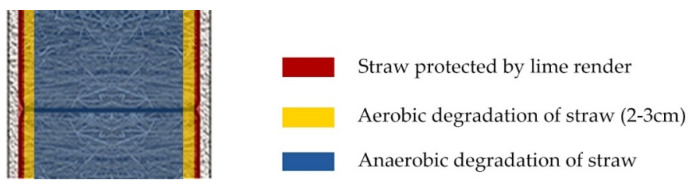
Analysis of straw degradation inside straw bale walls.

**Figure 15:**
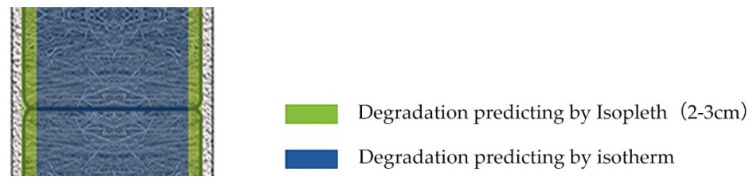
Critical factors for straw degradation inside straw bale walls.

The degradation between the lime render and the straw bales indicates the effect of aerobic degradation of straw. Due to the relatively high breathability of the lime render, the oxygen condensation behind the lime render would not be as low as the one in the straw bales. As a result, the aerobic degradation happens in the area behind the lime render in straw bales. However, due to the alkaline environment provided by the lime render, the degradation of straw is not serious with a mix of lime render. The degradation of straw was identified 2–3 cm behind the lime render. Because of the oxygen inside straw bales are much lower than the adjacent area of lime render and the straw bales, the degradation does not penetrate straw bales (Figure 14). However, if straw experiences serious degradation behind the lime render, hollows and cavity would likely form. Without the support of straw behind lime render, the render may have cracking issues and lead to water penetration into straw bales. As the isopleth model is designed to evaluate degradation levels of building materials exposed to the atmosphere [22], the model can be used to evaluate the straw conditions experiencing aerobic degradation behind the lime render. Due to the anaerobic degradation of straw being broadly dependent on the moisture content of straw, the existing isothermal models can predict the straw conditions inside walls (Figure 15).

## 6. Conclusions

The results of this research show moderate concerns of straw degradation between straw bales and the lime render regarding the climatic features in northern China. The experimental investigation shows that the hot and humid summer has insignificant impacts on the durability of straw bales within straw bale walls. The research has monitored a full-scale building in northeast China and compared it to laboratory scale results. This has resulted in lower degradation expectation of rice straw inside straw bale walls than the existing knowledge on the issue. In the small-scale, controlled conditions, rice straw has no notable degradation with two alignments in the 95% RH @ 35 °C for 12 weeks. Full scale building investigation identified limited degradation behind the render, whereas rice straw is in good condition with the protection of lime render and deep inside the walls. Due to the relatively high oxygen levels behind the lime render, the aerobic degradation is far more rapid than the anaerobic degradation, and the straw conditions against the lime render would be a concern pertaining to the durability of straw bale walls. Therefore, the isopleth models are suitable for predicting the straw degradation behind the rendering layer whereas the unsaturated isothermal model is more suitable for predicting degradation inside straw bale walls.

The work of this research builds on the understanding of the application of existing predicting models of straw degradation inside straw bale walls in the warm (humid) continental climate. The research identifies a limited degradation risk of rice straw bales inside a lime-based render layer with both the flat laying and the on-edge laying methods in the climatic condition of the warm (humid) continental climate. Further, straw bale buildings in this area will benefit from the render construction with high properties of moisture transmittance and low breathability. The results of the research justify the feasibility of straw bale building in resisting degradation with the climatic features of the warm (humid) continental climate. The impact of this research will be the growth in low-carbon energy efficient straw bale construction with confidence pertaining to its long-term durability characteristics.

## Figures and Tables

**Table 1:** Rendering thickness of opening locations and measured moisture.

Monitoring Location	Inner Monitored Moisture Content	Outer Monitored Moisture Content	Render Thickness
High position of west gable wall	20.3%	28%	75 mm
Low position of west gable wall	15.3%	24%	65 mm
High position of laid on-edge bale wall	12.8%	14.6%	50 mm
Low position of laid on-edge bale wall	17.3%	21.0%	70 mm
High position of east gable wall	18.0%	23.0%	105 mm
Low position of east gable wall	28.6%	33.0%	40 mm
High position of laid flat bale wall	17.0%	14.0%	50 mm
Low position of laid flat bale wall	24.0%	16.0%	100 mm

**Table 2:** Monitored moisture content and calculated moisture content from isothermal models.

Monitoring Location	Measured Moisture Content		Monitored RH	Yin et al. Equation [25]	Lawrence et al. Equation [24]
High position of west gable wall	Inner	20.3%	85.1%	19.4%	23.6%
	Outer	28%	90.5%	26.6%	32.1%
Low position of west gable wall	Inner	15.3%	74.9%	13.5%	16.4%
	Outer	24%	85.7%	20.0%	24.3%
High position of laid on-edge bale wall	Inner	12.8%	65.5%	10.5%	12.8%
	Outer	14.6%	71.7%	12.3%	15.0%
Low position of laid on-edge bale wall	Inner	17.3%	81.0%	16.5%	20.0%
	Outer	21.0%	83.7%	18.3%	22.2%
High position of east gable wall	Inner	18.0%	83.8%	18.4%	22.3%
	Outer	23%	86.8%	21.1%	25.6%
Low position of east gable wall	Inner	28.6%	91.9%	29.8%	36.0%
	Outer	33.0%	92.1%	30.3%	36.7%
High position of laid flat bale wall	Inner	17.0%	77.8%	14.7%	17.9%
	Outer	14%	72.7%	12.6%	15.4%
Low position of laid flat bale wall	Inner	24.0%	86.0%	20.3%	24.7%
	Outer	16%	82.6%	17.5%	21.2%
